# Lymphatic Territories (Lymphosomes) in a Canine: An Animal Model for Investigation of Postoperative Lymphatic Alterations

**DOI:** 10.1371/journal.pone.0069222

**Published:** 2013-07-24

**Authors:** Hiroo Suami, Shuji Yamashita, Miguel A. Soto-Miranda, David W. Chang

**Affiliations:** The Department of Plastic Surgery, The University of Texas MD Anderson Cancer Center, Houston, Texas, United States of America; University of KwaZulu-Natal, South Africa

## Abstract

**Background:**

Lymph node dissection is often performed as a part of surgical treatment for breast cancer and malignant melanoma to prevent malignant cells from traveling via the lymphatic system. Currently little is known about postoperative lymphatic drainage pattern alterations. This knowledge may be useful for management of recurrent cancer and prevention of breast cancer related lymphedema. We mapped the complete superficial lymphatic system of a dog and used this canine model to perform preliminary studies of lymphatic architectural changes in postoperative condition.

**Methods:**

Lymphatic territories (lymphosomes) were mapped with 4 female mongrel carcasses using an indocyanine green (ICG) fluorescent lymphography and a radiographic microinjection technique. Two live dogs were then subjected to unilateral lymph node dissection of lymph basins of the forelimb, and ICG lymphography and lymphangiogram were performed 6 months after the surgery to investigate lymphatic changes. Lymphatic patterns in the carcass were then compared with postoperative lymphatic patterns in the live dogs.

**Results:**

Ten lymphosomes were identified, corresponding with ten lymphatic basins. Postoperative fluorescent lymphographic images and lymphangiograms in the live dogs revealed small caliber lymphatic network fulfilling gaps in the surgical area and collateral lymphatic vessels arising from the network connecting to lymph nodes in the contralateral and ipsilateral neck in one dog and the ipsilateral subclavicular vein in another dog.

**Conclusion:**

Our canine lymphosome map allowed us to observe lymphatic collateral formations after lymph node dissection in live dogs. This canine model may help clarify our understanding of postoperative lymphatic changes in humans in future studies.

## Introduction

Breast cancer and malignant melanoma cells are well known to travel via the lymphatic system and migrate to regional lymph nodes. Thus, removal of regional lymph nodes has been a part of the surgical strategy for treatment of these cancers since Moore proposed axillary node dissection for the treatment of breast cancer in 1867 [1]. Sappey described the lymphatic system in the torso as being divided into 4 territories by the central midline and a horizontal line at the umbilical level, and he found that each lymph quadrant drained into the ipsilateral axillary or inguinal nodes in 1874 [2]. His anatomic findings have become the guiding principle in selecting which lymph basin needs to be removed in the treatment of a primary tumor [3].

Lymph node dissection can cause secondary lymphedema, which is a debilitating iatrogenic surgical complication. Radiation therapy increases the risk of developing lymphedema [4]–[5]. Little is known about the pathophysiology of lymphedema, and no standard of care has been established. The consensus among physicians is that obstruction of lymphatic pathways provokes lymph stasis in the affected limb and retention of protein-rich lymph fluid gradually induces fibrosis in the interstitial tissue [6]–[7]. However, there are no good explanations as to why some patients suffer from postoperative lymphedema and others do not.

Sentinel node biopsy has become a standard procedure used, when possible, to avoid the morbidity of radical lymph node dissection, including lymphedema, in the treatment of primary tumors [8]–[10]. Lymphatic preference dye and/or radioactive tracer are injected in the vicinity of the primary tumor to identify the first tier of lymph nodes. However, sentinel node biopsy cannot be used for the management of cancer metastasis caused by a locally recurrent tumor because previous treatment for the primary tumor and regional lymph node/s distorts the primary lymphatic drainage pattern.

Normal lymphatic pathways can be shown using lymphoscintigraphic examination during sentinel node biopsy. However, little information is available about postoperative lymphatic alterations. Such information, which could improve understanding of secondary cancer metastasis and of how to prevent lymphedema, would first require a comprehensive reference map of the lymphatic system at baseline to permit determination of where changes have occurred. Animal experiments are thought to be an essential element for this investigation, but, to the best of our knowledge, no standard, complete animal model of the lymphatic drainage system currently exists [11]–[12]. According to our previous research, the lymphatic system of a dog demonstrated remarkable similarities to the human lymphatic system in number, size, and distribution of the lymphatic vessels [13]–[14]. Thus, the aim of this study was to map the complete canine lymphatic system and demonstrate how this map can be used to better understand lymphatic architectural changes that occur after lymph node dissection.

## Materials and Methods

The animal protocol for this study was reviewed and approved by The University of Texas MD Anderson Cancer Center Institutional Animal Care and Use Committee, which is accredited by the Association for Assessment and Accreditation of Laboratory Animal Care International (Permit Number: 02-09-01972, 10-10-08371). All surgery was performed under isofulrane anesthesia, and all efforts were made to minimize suffering.

### Mapping Lymphosomes in Canine Carcasses

Four female mongrel hound carcasses weighing 23–30 kg were used for mapping lymphatic territories (lymphosomes). After the animals were euthanized, the carcasses were eviscerated and frozen at −30°C. Three bodies were cut into head and neck, forequarters, and hindquarters and the sections were investigated with radiographic microinjection technique. The remaining body was kept intact during the mapping and later cut in half lengthwise for comparison with postoperative images from a live dog that had undergone lymph node dissection.

**Figure 1:**
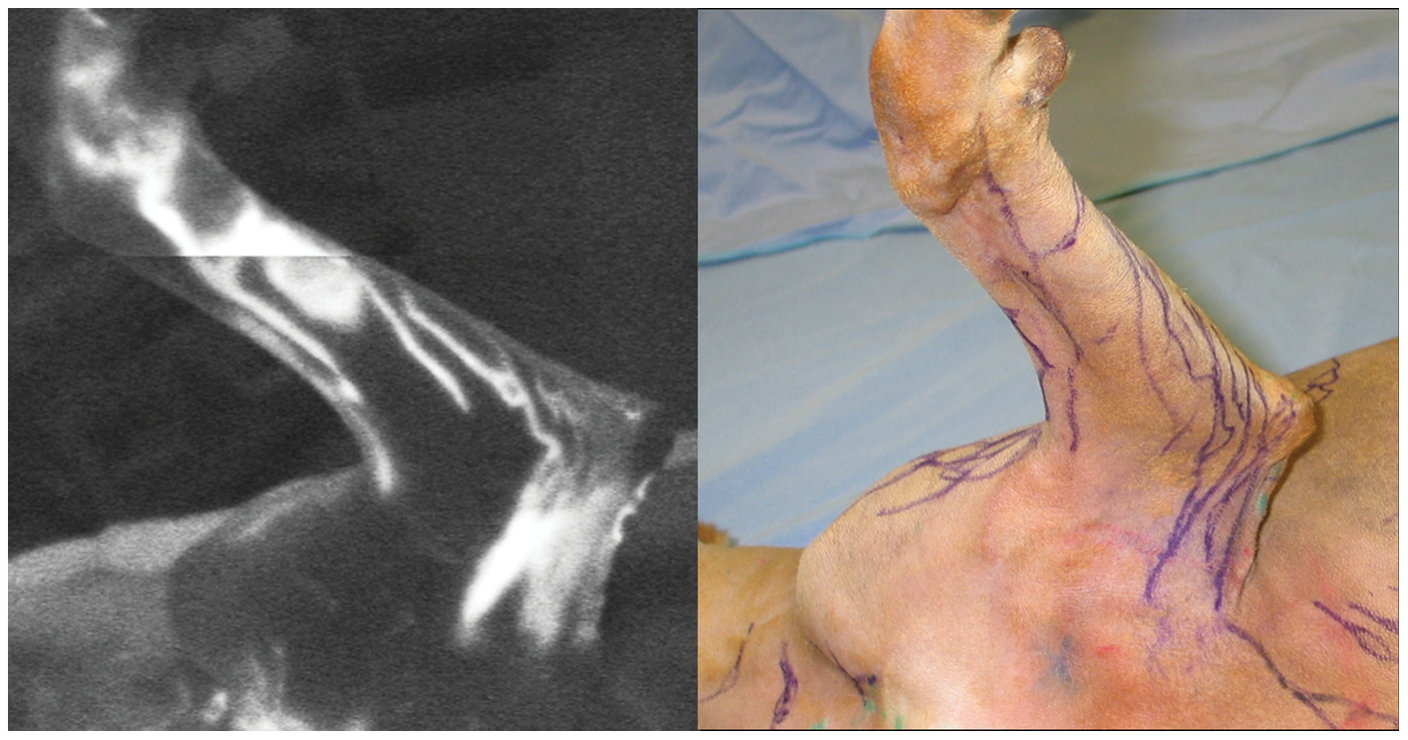
Indocyanine green (ICG) fluorescent lymphographic image of the medial side of the left forelimb in a dog carcass (left). Tracing of the lymphatic vessels visualized using ICG lymphography (right).

**Figure 2:**
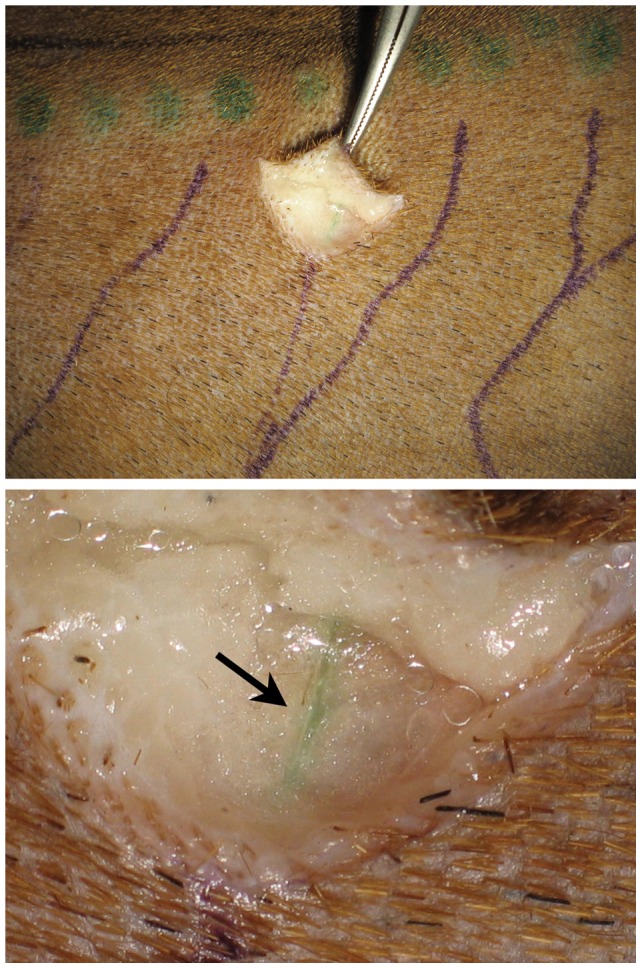
Marked lymphatic vessels near the dorsal midline in the torso (indocyanine green injection sites are shown as green dots) (top). A magnified photo shows that ICG was specifically taken into the lymph vessel (arrow) (bottom).

We used an indocyanine green (ICG) fluorescent lymphography system (PDE; Hamamatsu Photonics K.K., Hamamatsu, Japan) to locate lymphatic vessels in the whole-body carcass. First, 0.1 ml of ICG aqueous solution (IC-Green, 0.5 mg/ml; Akorn, Lake Forest, IL) was injected into the skin in multiple sites in the dorsal and ventral midline of the head and neck, torso, interdigital webspaces, and tail. The body was massaged gently to facilitate travel of the ICG inside lumen of the lymphatic vessel and then scanned with the ICG fluorescent lymphography system, and images were video-recorded. Finally, shiny lines visible on the monitor screen were traced on the skin with a marker (Fig. 1). These markings traced on the skin facilitated identification of the lymphatic vessels (Fig. 2).

**Figure 3:**
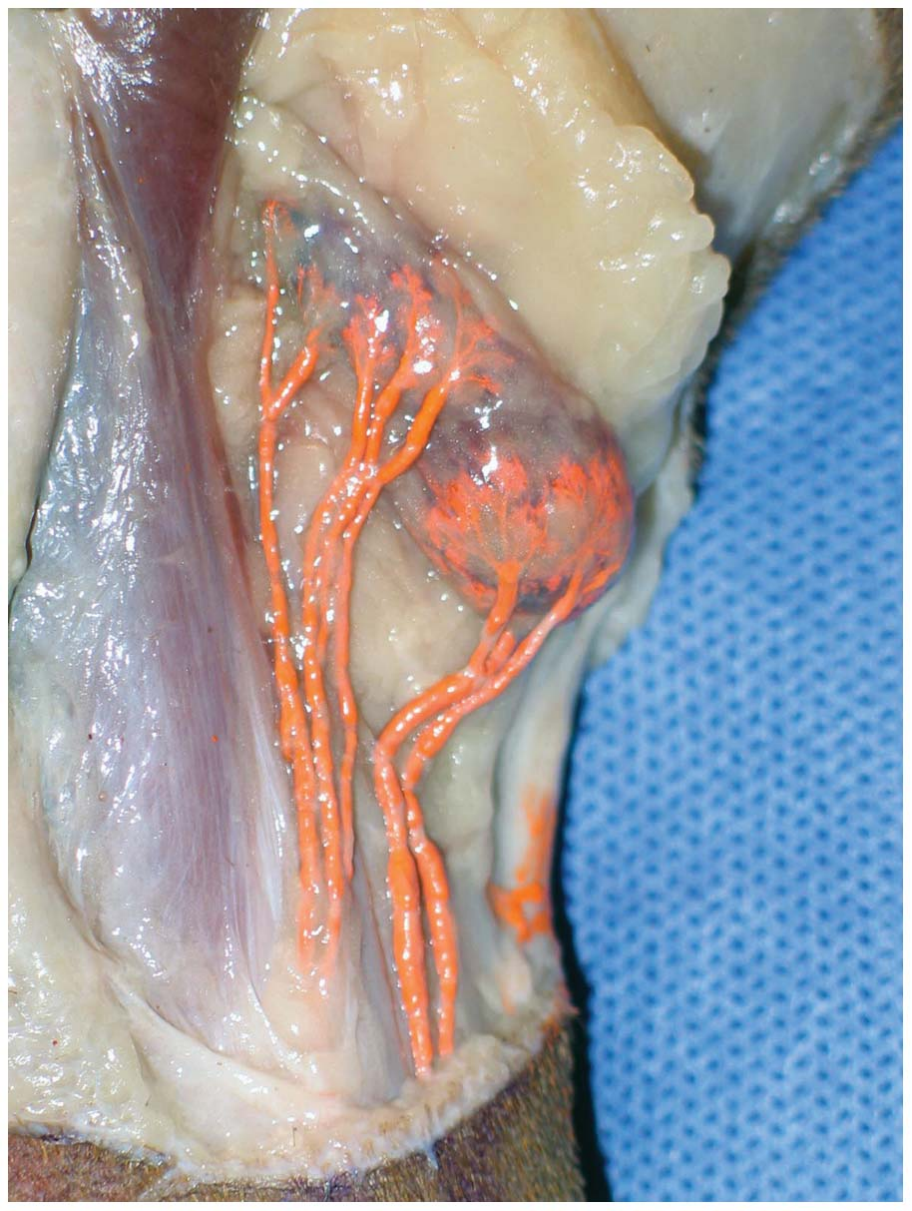
The popliteal lymph node with afferent lymphatic vessels (stained orange by the lead tetroxide radiocontrast mixture).

We used a radiographic microinjection technique that we had previously developed [15]–[16]. to investigate the canine lymphatic system in both the carcass sections and the whole body carcass following ICG lymphography. In brief, 3% hydrogen peroxide with 1% dye (Prussian Blue Professional Acrylic Ink; Liquitex Artists Materials, Piscataway, NJ) was injected into the skin in the search area. Fine oxygen bubbles produced from the hydrogen peroxide inflated the lymphatic vessel and forced the pigment into the lumen. A small incision was made 2.5 cm proximally from the injection site, and inflated lymphatic vessels were identified using a surgical microscope. A 30G 1-inch needle set with a micromanipulator (MN-153; Narishige International USA, Inc., East Meadow, NY) was then inserted into the lymphatic vessel, and an aqueous radiocontrast mixture (lead tetroxide; ScienceLab.com, Houston, TX) was injected manually using a 1-ml syringe. After injections to all lymphatic vessels were complete, the specimens were meticulously dissected until each lymphatic vessel connected to its corresponding lymph node (sentinel lymph node; Fig. 3). The specimen was radiographed in a digital format (FCR Go portable x-ray system; Fujifilm Medical Systems U.S.A., Inc., Stamford, CT).

**Figure 4:**
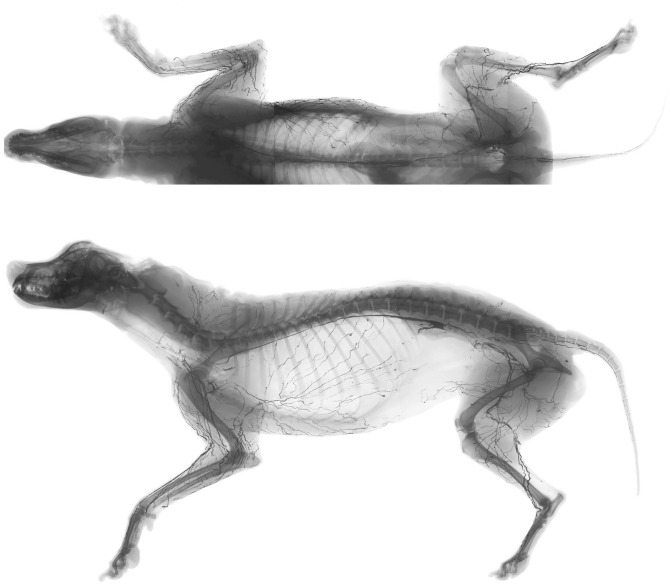
Anteroposterior (top) and lateral (bottom) radiographs of the whole body carcass after lymphatic injection.

Sections of the radiograph were composed in a montage using graphic software (Adobe Photoshop CS 5.5; Adobe Systems, Inc., San Jose, CA), and lymphatic vessels were traced (Fig 4). Each lymph node was color-coded in accordance with its regional lymphatic basin, and then each lymphatic vessel was color-coded retrogradely from its lymph node. Thus lymphatic territories (lymphosomes) were defined to reveal which area of the skin drained to which lymphatic basin.

### Lymph Node Dissection in a Dog

Two female dogs weighing 18 and 25 kg were used to investigate postoperative lymphatic changes after unilateral lymph node dissection. On the basis of our previous findings, the lymphatic system in the canine forelimb included 3 pathways: the dominant pathway to the ventral cervical node, the residual superficial pathway to the axillary node, and the deep pathway to the same axillary node approached from the cranial aspect [13]. The surgery attempted to disrupt all 3 pathways.

**Figure 5:**
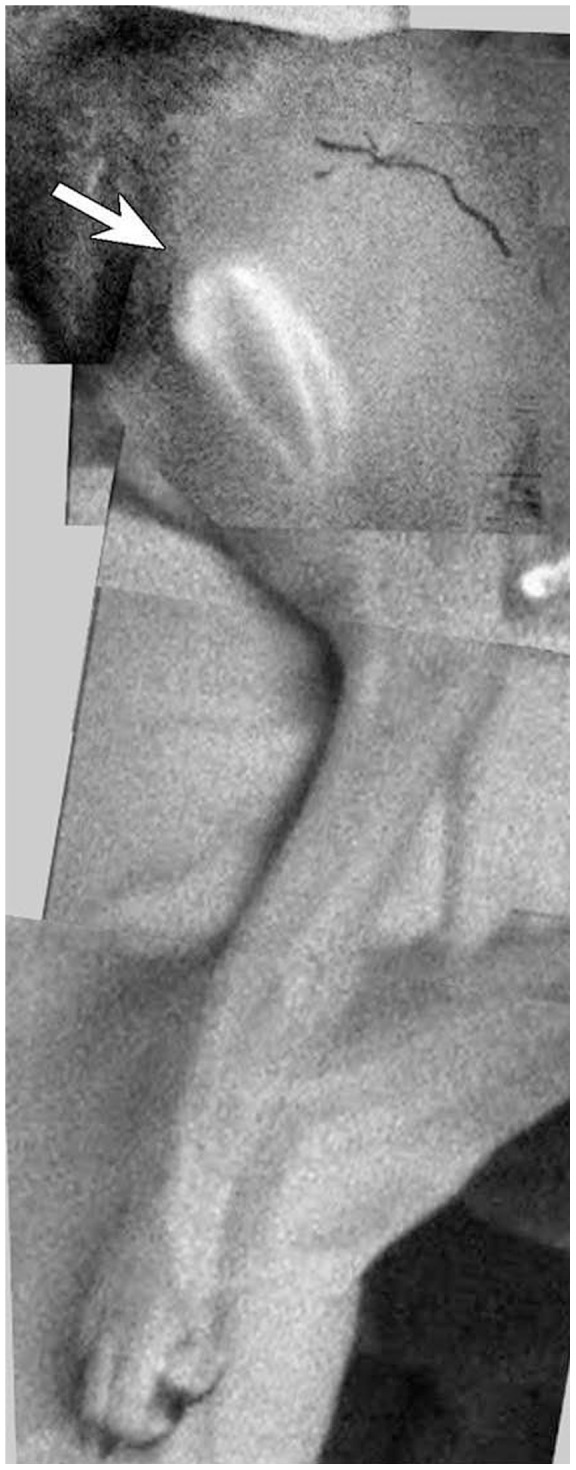
Montage of indocyanine green (ICG) lymphographic images of the left forelimb of the live dog prior to lymph node dissection. The ventral superficial lymph node (arrow) was identified using injections of ICG at interdigital webspaces.

After the dog was anesthetized with isoflurane, ICG fluorescent lymphography was used to demonstrate normal lymphatic pathways and accurately identify the locations of the lymph nodes prior to surgery (Fig 5). The ICG injection sites were either in interdigital webspaces in the forefoot – for locating the ventral cervical node – or on the medial side around the elbow joint, for locating the axillary lymph node. A skin incision of 10 cm was made above each lymph node. Isosulfan blue (Lymphozurin; Covidien, Mansfield, MA) was injected into the same sites as those used for ICG to stain the lymphatic vessels. Skin flaps were undermined with a very superficial layer because some lymphatic vessels ran immediately beneath the skin. Subcutaneous fat tissue, including the lymphatic vessels, was excised together with the underlying deep fascia and lymph node. Stamps of both the afferent and efferent lymphatic vessels were ligated or clipped. The skin incisions were closed with dermal and skin stitches, and penrose drains were inserted into the wound. Circumferential measurements of the operated forelimb at the paw, wrist, middle of the forearm, and elbow were recorded every other day for 3 weeks.

ICG lymphography and lymphangiography were performed 6 month after the surgery. After the dogs were anesthetized, ICG lymphography was performed as described in the previous section. For lymphangiography, isosulfan blue was injected into interdigital webspaces, a 4-cm incision in the dorsal paw was made, and a small cannula (Micro Cannulation System; Fine Science Tools Inc., Foster City, CA) was inserted into a stained lymphatic vessel and secured with 8–0 nylon. An oil-based radiocontrast agent (Lipiodol Ultra-Fuide; Guerbet, Cedex, France) was injected at a rate of 6 ml per hour using a syringe pump, and the forelimb and upper body were scanned with a C-Arm (Mobile C-Arm Series 9800; OEC Medical Systems, Inc., Salt Lake City, UT) and radiographed at 30-second intervals. These images were compared with the lymphosomes mapped on the carcasses to determine where the lymphatic system had changed.

Histological study was performed after sacrificing the dog with intravenous injection of Beuthanasia (1 ml/4.5 kg). The small cannula was inserted into a lymphatic vessel in the dorsal paw in the same manner as in lymphangiogram described above. Saline with 10% dye (Prussian Blue Professional Acrylic Ink; Liquitex Artists Materials, Piscataway, NJ) was injected manually with a 1 ml syringe. Resistance in the syringe signaled the end of injection. Several blocks of tissues including the skin, soft tissue, and superficial muscle were harvested from the stained area, fixed in 10 % formalin, and stained with hematoxylin and eosin (HE).

## Results

### Lymphosomes in a canine

We found that ICG fluorescent lymphography could detect lymphatic vessels even though the specimens had been frozen and thawed, and that ICG could actively travel through the vessels in the absence of live smooth muscle. Although the ICG traveled only halfway to each lymph node from the injection site, it was specifically taken into the lymph vessels, which allowed us to identify them using markings on the skin, thus facilitating microinjection of the radiocontrast mixture to verify the locations of the vessels. Imaging in the canine carcasses showed that the lymph vessels emerged 3–5 cm from the dorsal and ventral midline. Only lymph capillaries in the dermis were observed near the midline area; no lymphatic vessels were found to cross the midline in either the ventral or dorsal aspects.

**Figure 6:**
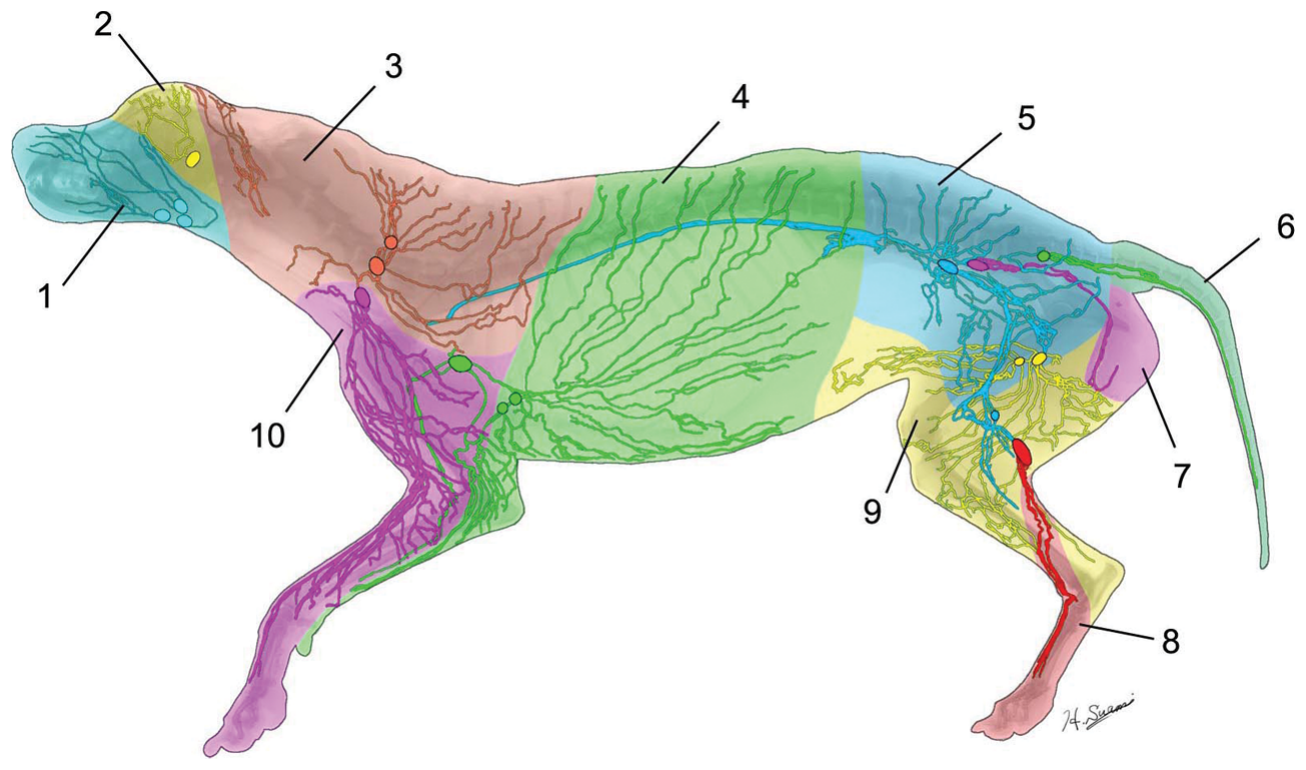
Color-coded diagram of the lymphatic territories (lymphosomes) with lymphatic vessels shown distally from their corresponding lymph nodes: 1, submandibular; 2, parotid; 3, dorsal superficial cervical; 4, axillary; 5, medial iliac; 6, lateral sacral; 7, hypogastric; 8, popliteal; 9, superficial inguinal; 10, ventral superficial cervical.

Ten lymphatic basins were identified in the canine body: submandibular, parotid, dorsal superficial cervical, axillary, medial iliac, lateral sacral, hypogastric, popliteal, superficial inguinal, and ventral superficial cervical (Fig. 6). The anatomic locations of the lymphatic basins were consistent among the specimens; however, the number of lymph nodes in each basin varied from 1 to 3. No overlapping of the superficial lymphatic vessels was observed except in the head region. In the head region, the vessels ran in 2 different layers, which were above and below the facial muscles. The lymphatic vessels were interconnected within the same nodal basin but not with the neighboring territory. Therefore, one lateral half of the dog skin could be divided into 10 lymphosomes.

### Changes in the Lymphatic System after Lymph Node Dissection

After the initial surgery in both dogs, the skin flap in the cervical area developed wound dehiscence with marginal necrosis, which required revision on postoperative day 3 and 7 in each dog after specification of demarcation line. There were no further complications, and the dog was maintained in stable condition thereafter. The forelimbs on the operated side gradually swelled starting on the third day after the initial surgery, and this swelling reached peaks around 10 days; the circumferential measurements at the elbow were 18.1% and 14.5 % larger than the preoperative elbow measurements. The swelling in the forelimbs gradually subsided and completely disappeared 3 weeks after the initial surgeries.

**Figure 7:**
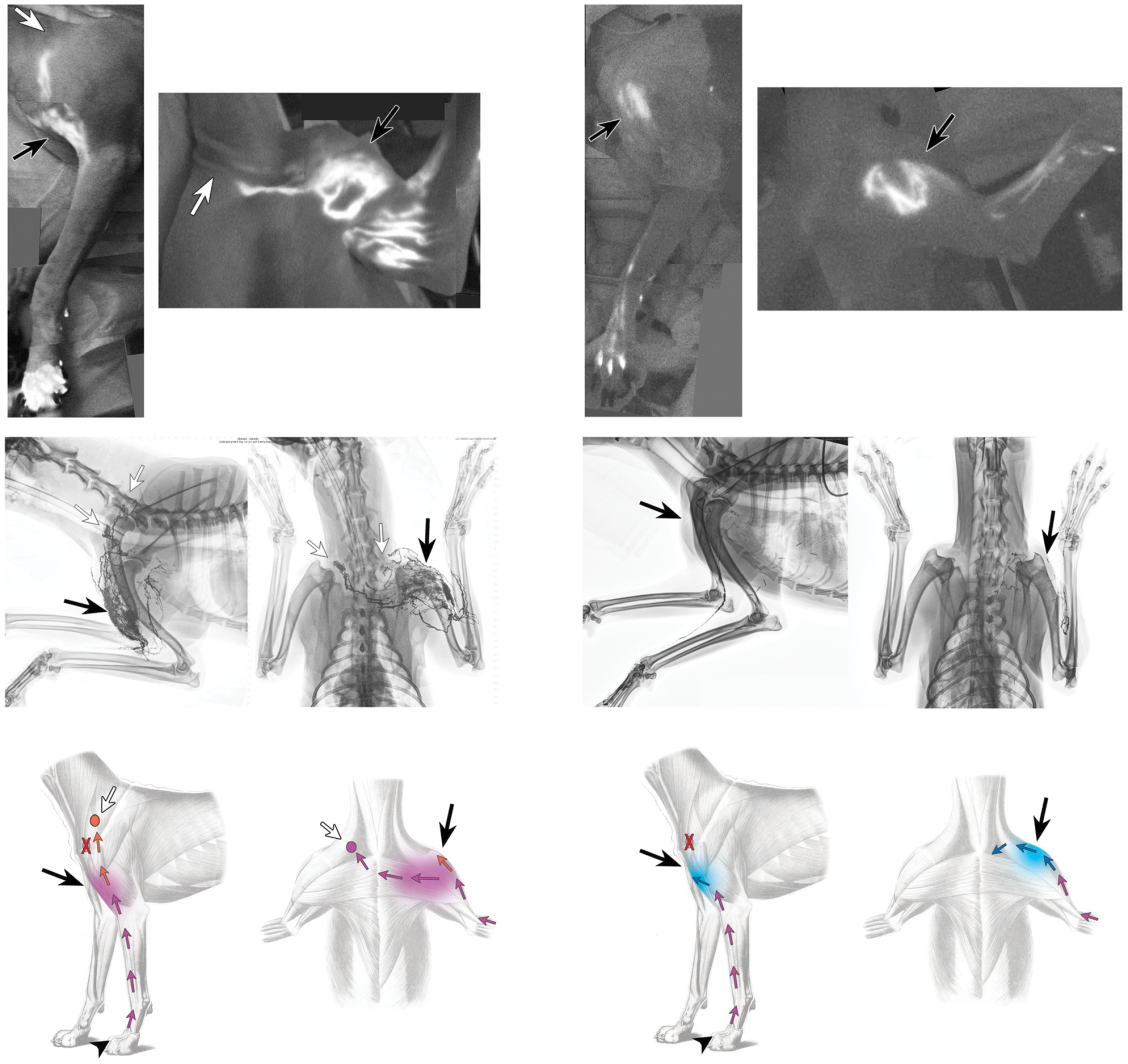
A montage of indocyanine green lymphographic images of the left forelimbs of 2 live dogs 6 months after lymph node dissection (top). Bright spots were seen in the area in which the surgery took place (black arrow). Locations of lymph nodes are marked (white arrows). Lymphangiograms from the same dogs from lateral (left) and antero-posterior (right) views showing capillary-like network (black arrows) and bypassed lymph nodes (white arrows) (middle). Diagrams show changes of lymphatic pathways (bottom).

The postoperative ICG lymphographic images showed a wide, spotty blight area where the surgery took place (Figure 7, top row, Video S1). In the first dog, two unusual lymphatic pathways were found proximal to the area in which the surgery took place. The first pathway ran toward the ventral superficial cervical lymph node, and the other pathway crossed the ventral midline and connected to the contralateral ventral superficial cervical node. In the second dog, we could not chase any lymphatic vessels from the blight area.

Lymphangiograms revealed similar findings, along with precise, high-resolution chronological information about radiocontrast media movement. The lymphatic vessels looked normal from the dorsal side of the forelimb to the area in which the surgery took place, but they then diverged and formed a capillary-like network. These tiny vessels gradually converged to form a few lymphatic vessels that connected to the adjacent lymph basins in the first dog and to the subclavicular vein in the second dog. (Figure 7, middle and bottom rows, Video S2). These vessels were functioning as collateral drainage pathways from the affected forelimb after obstruction of the normal pathways.

**Figure 8:**
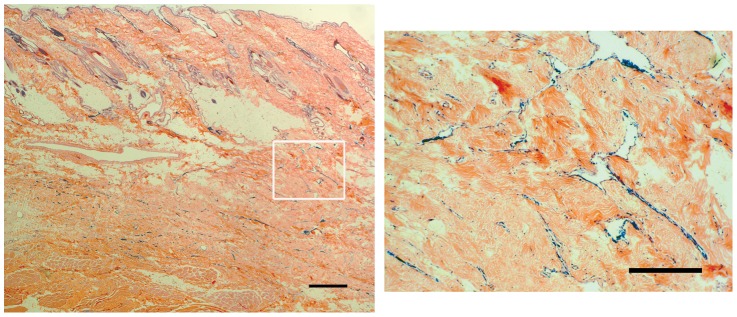
Histological image with hematoxylin and eosin staining of a cross section of a skin in the capillary-like network (scale bar: 5 mm) (left). Small lymphatics lined with pigment is found in the entire fibrous scar tissue (scale bar: 2 mm) (right: white square field of the left).

Histological image demonstrated that pigments of the injected dye remained in the lumen of the capillary-like network, and thus enabled lymphatics to be distinguished. (Fig. 8). Cross section revealed that these lymphatic capillaries were situated extensively inside fibrous tissue within the surgical scar.

## Discussion

Our radiographic microinjection technique allowed us to create a comprehensive lymphatic topography in a dog carcass, and we found that the canine lymphatic system contained 10 lymphatic territories (lymphosomes). We were then able to use this information to show how the structure of lymphatic vessels changed in live dogs after forelimb lymph node dissection.

Knowledge of the anatomy of the lymphatic system, both in animals and in humans, is very limited [16]–[18]. Baum and Ellenberger investigated and described the lymphatic system in domestic animals, including the horse, cow, pig, dog, and chicken [19]–[22]. Their findings for the dog were precise and similar to ours, but no photographic or imaging data from their study are available for comparison. Mapping of lymphosomes – dividing the body into territories showing each lymph node basin, as we have done in this study – is a new concept that we recently introduced [13]. Lymphosomes can be used to determine which lymph basins should be dissected on the basis of the primary tumor location, and they can also be used as a control for analysis of postoperative lymphatic changes.

**Figure 9:**
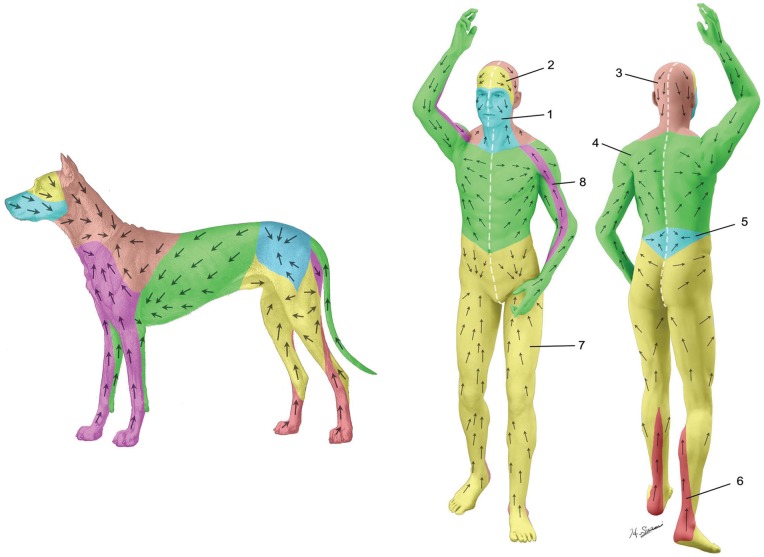
Human lymphosomes, similar to the canine lymphosomes. 1, Frontal cervical; 2, parotid; 3, posterior cervical; 4, axillary; 5, retroperitoneal; 6, popliteal; 7, superficial inguinal; 8, subclavicular.

Using previously published information about human anatomy in the superficial lymphatic system [2], [23]–[28], as well as information from our own dissections [29]–[32] and from lymphoscintigraphic examinations in clinical settings [33]–[36], we created a tentative human lymphosome map (Fig. 9). The sizes of each territory were discordant with those of the canine lymphosomes, but otherwise the 2 diagrams demonstrated remarkable similarities. Thus, a canine model such as the one used in the present study may be a good animal model for human lymphatic studies. This human lymphosome map is preliminary and will need to be refined by further anatomic investigation, but it may provide a prospective blueprint for cancer management.

The postoperative ICG lymphographic images and lymphangiograms of the live dogs in our study showed that lymphatic vessels in the obstructed territory connected to the lymph nodes in adjacent territories and spontaneous lymphaticovenous shunt. These collaterals are thought to act as bypasses to prevent manifestation of lymphedema and new metastatic pathways of residual cancer. The timing of the collateral formation in our study was uncertain, but the reduction of limb size suggests that this process occurred within 3 weeks after the surgical obstruction of the vessels. The capillary-like network played a key role to form the collaterals and this may develop together with scar formation.

A lymph vessel crossing the midline, as we observed in the live dog after lymph node dissection, has not been observed in previous examinations of the canine lymphatic system [13]–[14], [21], nor did we observe it in our examination of canine carcasses in this study; the collateral vessels went in the opposite directions until passing the sternum. The rerouted vessels in the live dog are reminiscent of the process of vascular remodeling in flap surgeries, in which choke vessels are dilated and then linked to adjacent territories to compensate for the lost vascular supply in the source territory [37]–[38]. However, unlike in the vascular system, the collecting lymphatic vessel contains bicuspid valves in its lumen at very short intervals. Therefore, it is unlikely that the collateral vessels found in this study were an altered form of pre-existing lymphatic vessels with incompetent valves. We hypothesize that lymph fluid was rerouted superficially toward the lymph capillary network in the dermis when the collecting lymphatic vessel became obstructed by surgery. Lymph retention in the capillary may stimulate lymphangiogenesis, and new lymphatic collector vessels may be created from the dilated capillary. These collateral vessels sprout from the capillary network and grow toward the nearest escape route.

**Figure 10:**
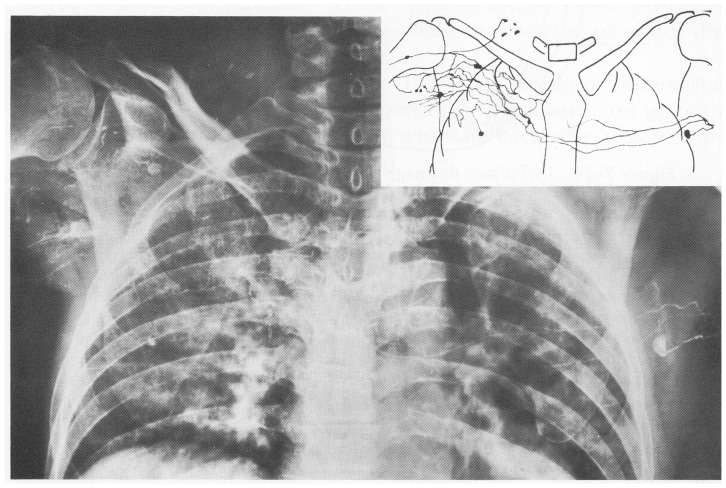
Postoperative lymphangiogram of a patient who underwent right breast mastectomy and axillary node dissection. Injected radiocontrast from the affected hand reached the contralateral axillary and ipsilateral supraclavicular nodes. Compare with the lymphangiogram of the live dog in Fig. 7. (Reprinted with permission from Bobbio P, Peracchia G, Pellegrino F. (1962) Connessioni linfatiche presternali fra le regioni mammarie dei due lati. *Ateneo Parmensa*, 33(supp.): 95–109).

A study did report this phenomenon in a human patient. In the article describing lymphangiographic findings in patients who had undergone mastectomy and lymph node dissection, radiocontrast media injected into the arm nearest to where the dissection had occurred reached the ipsilateral axillary lymph node via a network structure in the ipsilateral axillary region and via the collecting lymphatic vessel, which crossed the front midline (Fig. 10) [39]. This image is similar to the postoperative lymphangiogram of the first live dog. Aboul-Enein et al. reported that spontaneous lymphovenous shunt was observed in non-edematous arm by lymphangiogram in 2 patients out of 20 who underwent radical mastectomy and axillary node dissection [40].

Sentinel node biopsy in patients undergoing postoperative axillary surgery has been described in several articles [41]–[45]. In 11 (8%) of 135 patients who underwent reoperative sentinel node biopsy with successful mapping, the sentinel node was the contralateral axillary lymph node, and in 6 patients (4%), the ipsilateral clavicular node was the sentinel node. These reports speculate that collateral lymphatics were formed in patients after the axillary surgery and lymphatic pathways altered its course to adjacent lymphatic territories similar to our live dog study.

A better understanding of postoperative lymphatic changes may help physicians predict secondary cancer metastasis and improve their understanding of the pathophysiology of postoperative lymphedema. It remains unclear, for example, why some patients suffer from lymphedema after axillary node dissection and others do not. We believe that a canine model and the lymphosomes concept can help lead to new insights into the lymphatic system.

## Conclusions

We have successfully mapped canine lymphosomes and showed how this map can be applied to determine lymphatic changes following lymph node dissection. Because many similarities have been identified between lymphosomes in dogs and humans, this canine model may be of use in future studies of the human lymphatic system, particularly in determining postoperative lymphatic alterations. Postoperative fluorescent lymphographic images and lymphangiograms of the live dog showed collateral lymph formation. These preliminary findings may provide useful information for future studies aimed at developing methods to predict secondary cancer metastasis and prevent secondary lymphedema.

## Supporting Information

Video S1 **Indocyanine green lymphographic image of the left forelimbs of a live dog 6 months after lymph node dissection.**
(WMV)

Video S2 **Lymphangiogram from the same dog from antero-posterior view showing capillary-like network and collateral pathways.**
(WMV)
